# Cardioish: Lead-Based Feature Extraction for ECG Signals

**DOI:** 10.3390/diagnostics14232712

**Published:** 2024-11-30

**Authors:** Turker Tuncer, Abdul Hafeez Baig, Emrah Aydemir, Tarik Kivrak, Ilknur Tuncer, Gulay Tasci, Sengul Dogan

**Affiliations:** 1 Department of Digital Forensics Engineering, Technology Faculty, Firat University, 23200 Elazig, Turkey; turkertuncer@firat.edu.tr; 2 School of Management and Enterprise, University of Southern Queensland, Toowoomba, QLD 4350, Australia; abdul.hafeez-baig@unisq.edu.au; 3 Department of Management Information Systems, Management Faculty, Sakarya University, 54050 Sakarya, Turkey; emrahaydemir@sakarya.edu.tr; 4 Department Cardiology, Firat University Hospital, Firat University, 23200 Elazig, Turkey; tkivrak@firat.edu.tr; 5 Elazig Governorship, Interior Ministry, 23100 Elazig, Turkey; ilknur.tuncer@icisleri.gov.tr; 6 Department of Psychiatry, Elazig Fethi Sekin City Hospital, 23100 Elazig, Turkey; akcagulay01@gmail.com

**Keywords:** Cardioish, symbolic language, feature extraction, machine learning

## Abstract

**Background:** Electrocardiography (ECG) signals are commonly used to detect cardiac disorders, with 12-lead ECGs being the standard method for acquiring these signals. The primary objective of this research is to propose a new feature engineering model that achieves both high classification accuracy and explainable results using ECG signals. To this end, a symbolic language, named Cardioish, has been introduced. **Methods:** In this research, two publicly available datasets were used: (i) a mental disorder classification dataset and (ii) a myocardial infarction (MI) dataset. These datasets contain ECG beats and include 4 and 11 classes, respectively. To obtain explainable results from these ECG signal datasets, a new explainable feature engineering (XFE) model has been proposed. The Cardioish-based XFE model consists of four main phases: (i) lead transformation and transition table feature extraction, (ii) iterative neighborhood component analysis (INCA) for feature selection, (iii) classification, and (iv) explainable results generation using the recommended Cardioish. In the feature extraction phase, the lead transformer converts ECG signals into lead indexes. To extract features from the transformed signals, a transition table-based feature extractor is applied, resulting in 144 features (12 × 12) from each ECG signal. In the feature selection phase, INCA is used to select the most informative features from the 144 generated, which are then classified using the k-nearest neighbors (kNN) classifier. The final phase is the explainable artificial intelligence (XAI) phase. In this phase, Cardioish symbols are created, forming a Cardioish sentence. By analyzing the extracted sentence, XAI results are obtained. Additionally, these results can be integrated into connectome theory for applications in cardiology. **Results:** The presented Cardioish-based XFE model achieved over 99% classification accuracy on both datasets. Moreover, the XAI results related to these disorders have been presented in this research. **Conclusions:** The recommended Cardioish-based XFE model achieved high classification performance for both datasets and provided explainable results. In this regard, our proposal paves a new way for ECG classification and interpretation.

## 1. Introduction

Cardiovascular diseases (CVDs) are a major health problem worldwide [1,2]. This has led to the need for effective and reliable diagnostic methods [3]. One of the most widely used techniques for monitoring heart activity is electrocardiography (ECG), which offers a non-invasive way to detect various heart conditions by monitoring the heart’s electrical signals [4]. Traditionally, approaches to ECG analysis have emphasized classification accuracy, with deep learning (DL) models being a popular choice [5]. However, while these models tend to perform well, they often come with the disadvantage of complexity, and their results can be difficult for clinicians to interpret; this is especially important in medical settings [6,7].

In recent years, the focus has shifted towards explainable artificial intelligence (XAI) [8,9,10]. The aim is to make machine learning models more transparent so that healthcare professionals can better understand and trust the results they provide [11]. This has led to the development of newer methods that aim to balance strong performance with a level of interpretability that is useful in everyday medical practice [12].

The Cardioish model provides a new perspective on ECG analysis. It offers a symbolic language that not only achieves high classification accuracy but also facilitates the interpretation of results. By converting the features of ECG signals into simpler symbols, this model aims to make the data more understandable for clinicians. In addition, by integrating the connectome theory [13], the approach provides not only diagnostic predictions but also some insights into the physiological processes behind different heart conditions.

The Cardioish approach attempts to address some of the current challenges facing machine learning in cardiology. By combining simple feature extraction methods with a symbolic language, the model aims to offer a balance between accuracy and ease of interpretation. The ultimate goal is to contribute to more accessible and reliable tools for diagnosing heart conditions, presenting the information in a way that is easy for healthcare providers to visualize and understand.

### 1.1. Literature Review

Different diseases have been detected using ECG in the literature. Some of these studies are presented below.

Yoon et al. [14] proposed a bimodal CNN model to classify cardiovascular diseases using ECG grayscale images and scalograms. Using a dataset of 10,588 ECG recordings, their model co-trained both image types with Inception-v3 networks. The model achieved 95.08% accuracy and 99.20% AUC for lead II. Li et al. [15] presented a deep learning method using ECG data from the MIT–BIH arrhythmia database. By applying Empirical Mode Decomposition (EMD) and classifying 2D feature maps with a CNN, their model achieved a 99.01% accuracy. Goud et al. [16] developed a Wolf-based Generative Adversarial System (WbGAS) for heart disease classification using ECG data. Their model achieved 99.86% accuracy, with 99.41% precision and 99.13% recall. Sharma et al. [17] suggested using wavelet scattering on ECG signals to identify sleep disorders from the CAP sleep database. Their method achieved an overall accuracy of 98%, with up to 99.65% accuracy for specific disorders using the ensemble bag of trees classifier. Loh et al. [18] proposed a deep neural network model for attention deficit hyperactivity disorder and conduct disorder detection using ECG data. Their model, trained on 123 participants, achieved 96.04% accuracy and used Grad-CAM for explainable feature analysis. Baygin et al. [19] presented an ECG-based model for automated anxiety detection using data from 19 subjects. Their model, employing probabilistic binary patterns and wavelet transforms, achieved over 98.5% accuracy in classifying anxiety levels. Khare et al. [20] developed ECGPsychNet, a hybrid ensemble model for detecting psychiatric disorders using ECG data from 233 subjects. The model achieved 98.15% accuracy, with detection rates of 98.96% for schizophrenia, 96.04% for depression, and 95.12% for bipolar disorder. Malakouti et al. [21] proposed an approach for heart disease classification using ECG data from 302 individuals. Their approach achieved the highest accuracy at 96%. Parveen et al. [22] suggested a one-dimensional residual deep convolutional auto-encoder model for heart disease classification using the MIT–BIH arrhythmia dataset. Their model achieved 99.9% accuracy and 99.8% specificity. Lee and Kim [23] proposed a vehicle-embedded ECG system using single-lead signals. They used 8528 ECGs from the PhysioNet 2017 dataset and a two-stage machine learning model. The system achieved an F1 score of 78.98% with real-time classification in 0.86 s. Karapinar Senturk et al. [24] presented a deep learning model that converts ECG signals into scalogram images using continuous wavelet transform. Using PhysioNet data, the model achieved 98.67% accuracy in classifying arrhythmia, congestive heart failure, and normal sinus rhythm. Qin et al. [25] proposed the multi-view knowledge transferring-ECG model to improve single-lead ECG classification using knowledge transfer from multi-lead ECGs. Tested on PTB-XL and ICBEB2018 datasets, the model achieved performance improvements of 1.3% and 3.2%, respectively. Han et al. [26] proposed a multimodal learning model for long-term ECG classification using ECG signals and gramian angular field images. Their model achieved over 86% F1 scores on the St. Petersburg INCART and MIT–BIH Supraventricular datasets. Narotamo et al. [27] compared deep learning methods for ECG classification using the PTB–XL dataset. Their study found that the gated recurrent unit model, applied to 1D ECG signals, performed best with 79.67% sensitivity and 81.04% specificity. Singhal et al. [28] proposed a method using high-resolution superlet transform and VGG19 for classifying ECG and PCG signals. Using the PhysioNet/CinC 2016 database, their model achieved 93.1% accuracy for ECG and 82.1% for PCG. Nawaz et al. [29] proposed the COVID–ECG–RSNet model using ECG images for COVID-19 classification. Using 1937 images, the model achieved 98.8% accuracy, with 98.9% precision and 98.7% recall. Arslan et al. [30] developed a dilated convolutional autoencoder for ECG classification using the MIT–BIH dataset, achieving 99.99% accuracy by simultaneously training feature extraction and classification. Choudhury et al. [31] presented an exponential political optimizer-trained deep quantum neural network model for arrhythmia detection using the MIT–BIH databases, achieving 91.4% accuracy, 92% sensitivity, and 91.7% specificity. Abagaro et al. [32] proposed an ECG classification system using the MIT–BIH dataset, employing discrete wavelet transform, principal component analysis, and adaptive neuro-fuzzy inference system. Their model achieved 99.44% accuracy, with 99.36% sensitivity and 99.84% specificity. Sadad et al. [33] proposed a lightweight CNN with an attention module for ECG classification, achieving 98.39% accuracy using a 12-lead ECG dataset with various cardiac conditions. Kim et al. [34] suggested an ECG stitching scheme for arrhythmia classification using data from the PhysioNet/CinC Challenge 2017. Their CNN model achieved 82.39% accuracy on stitched ECG data, compared to 88.99% on the original data.

### 1.2. Literature Gaps

The detected literature gaps are as follows:

- Due to their high classification performance, many researchers have turned to deep learning (DL) models. However, DL models come with high computational complexities.
- Connectome theory is primarily associated with brain-related models. To our knowledge, there are no existing connectome projects or research efforts in cardiology.
- Most machine learning-based ECG classification models have focused solely on classification performance. There is a lack of work on explainable artificial intelligence (XAI) and cardiology in the literature.

### 1.3. Motivation and Our Model

Our main motivation is to introduce the Cardioish symbolic language to integrate connectome theory into cardiology. Firstly, we need a lightweight model because most existing models use deep learning (DL) approaches, which have high time complexity. Although feature engineering models tend to have relatively lower classification performance, we address this gap by presenting a new approach inspired by transformers. Therefore, we have proposed a novel lead transformer technique to convert ECG signals into lead identifiers. To extract hidden patterns and unseen transitions, we developed a transition table-based feature extractor, which, together with the lead transformer, extracts 144 features.

In the feature selection phase, an iterative feature selection method has been employed. Specifically, the iterative neighborhood component analysis (INCA) has been used to choose the most informative features. These selected features have been used to generate both classification and explainable results.

To demonstrate the high classification capability of the proposed Cardioish-based XFE model, we employed the k-nearest neighbors (kNN) classifier.

As stated in the literature, there are relatively few XAI-based biomedical signal classification models, and most existing XAI models rely on well-known techniques. To address this gap, we have proposed a new symbolic language called Cardioish. Cardioish consists of 12 symbols, each representing a different lead. By using this language, we aim to integrate connectome theory into cardiology using easily collected biological signals.

### 1.4. Novelties and Contributions

Novelties:

- We have proposed a new transformation method named the “lead transformer”. By using this transformation, the effects of the leads have been extracted.
- Cardioish, a new symbolic language, has been introduced, allowing explainable results to be generated from ECG signals.
- A new XFE (Explainable Feature Engineering) model has been presented, utilizing the recommended Cardioish language.
- By employing Cardioish, connectome theory has been integrated into cardiology.

Contributions:

- The proposed XFE model has significantly contributed to ECG signal classification, achieving over 99% classification accuracy on the utilized ECG beat datasets. The high classification accuracy across two datasets demonstrates the general applicability of the presented model.
- Cardioish has been introduced to generate interpretable results from ECG signals. By leveraging the findings from the proposed Cardioish, connectome theory has been integrated into cardiology. Furthermore, this integration has the potential to inspire the proposal of new explainable theorems, further contributing to the field of cardiology.

## 2. Materials and Methods

### 2.1. Material

In this research, two distinct ECG beat datasets were used, each containing 12 leads with a length of 651 for all datasets. These datasets are (i) the MI (Myocardial Infarction) dataset and (ii) the mental disorder detection dataset. The details of these datasets are provided below.

#### 2.1.1. MI Dataset

The first dataset used is the public MI ECG beat dataset [35], which contains 10 types of myocardial infarctions (MIs) and a control group, totaling 11 classes. This dataset was downloaded from PhysioBank. It includes 49,235 ECG beats with the following distribution: (0) Healthy—10,305 beats, (1) Anterior—4659 beats, (2) Anterior Lateral—6142 beats, (3) Anterior Septal—7976 beats, (4) Inferior—10,215 beats, (5) Inferior Lateral—5822 beats, (6) Inferior Posterior—48 beats, (7) Inferior Posterior Lateral—2495 beats, (8) Lateral—459 beats, (9) Posterior—459 beats, and (10) Posterior Lateral—655 beats. This dataset is commonly used in machine learning research for detecting MI types, which is why it was selected for this study.

#### 2.1.2. EEG Stress Dataset

We used this dataset to detect the cardiac effects of mental disorders on ECG signals. The dataset was collected from 198 participants with mental disorders, distributed as follows: 62 with bipolar disorder, 17 with depression, and 119 with schizophrenia (SZ). Additionally, ECG beats from participants with no findings were included in the dataset. In total, there are 841 ECG beats for bipolar disorder, 202 for depression, 1627 for SZ, and 900 for healthy individuals [36].

### 2.2. The Proposed Cardioish-Based Explainable Feature Engineering Model

The main objectives of the proposed Cardioish-based XFE model are:

- Attaining high and general classification performance,
- Proposing a model with linear time complexity,
- Presenting XAI results.

Therefore, we have proposed the Cardioish-based XFE model, which consists of four main phases:

- Feature extraction: In this phase, the presented lead transformer and transition table-based feature extractor are used. A total of 144 features are extracted.
- Feature selection: To meticulously select the most informative features, we have used the INCA feature selector, which is applied to the 144 generated features.
- Classification: The kNN classifier is employed to obtain classification results, demonstrating the high classification ability of the selected features.
- XAI: In this phase, we use the recommended Cardioish language. By using the selected features, Cardioish symbols are extracted, and a Cardioish sentence is created. Subsequently, connectome graphs of the generated Cardioish sentence are created, which are used to identify cardiac situations.

**Figure 1:**
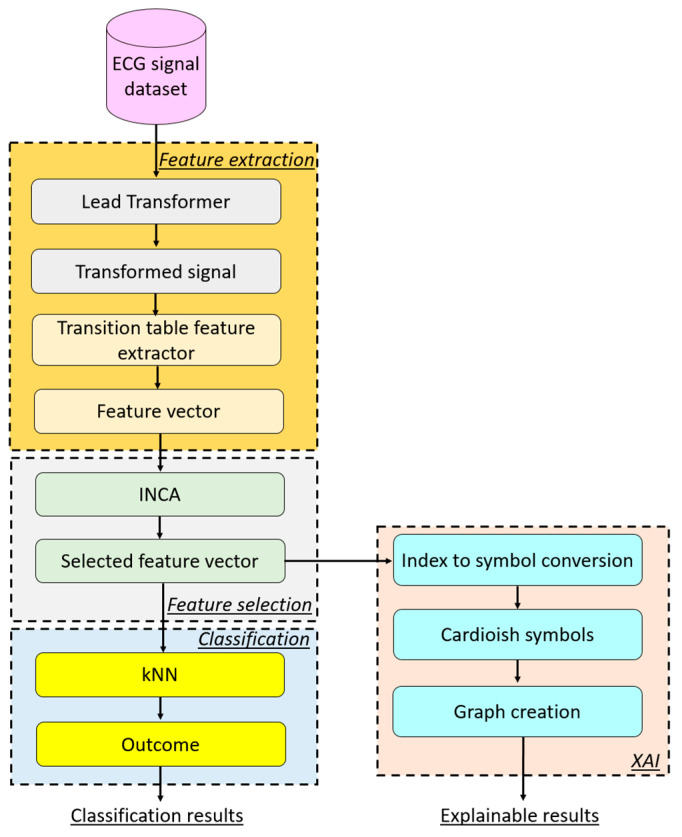
Overview of the proposed Cardioish-based XFE model.

To better explain the proposed model, a graphical overview of this model is shown in Figure 1.

The details of the proposed Cardioish-based XFE model are provided below.

#### 2.2.1. Feature Extraction

The first phase of the presented model is the feature extraction phase. To generate features, a lead transformer and a transition table pattern-based feature extractor were used. By applying the proposed lead transformer, the ECG signals were converted into lead identities, and features were extracted based on the transitions between these identities. Since the ECG beat datasets used contain 12 leads, the proposed feature extraction function generated 144 (12 × 12) features. The steps of the proposed model are as follows:

Step 1: Apply the proposed lead transformer to each ECG lead and obtain the transformed signal.

(1) $$bl\left(1:12\right)=ecg\left(1:12,i\right),\;i\in \{1,2,\ldots ,len\}$$

(2) $$[sorted,id]=argsort\left(-bl\right)$$

(3) $$tr\left(c:c+11\right)=id,\;c\in \{1,13,\ldots ,12\times len\}\;$$

Herein, bl: lead block, ecg: ECG signal, argsort: sorting function, len: length of the signal and tr: transformed signal. In this transformation, sorting by descending order based on the ECG was used to identify the lead. 

Step 2: Extract 144 features by deploying transition table-based feature extraction. We have used this feature extraction function to detect the used leads.

(4) $$mat\left(id\left(j\right),id\left(j+1\right)\right)=mat\left(id\left(j\right),id\left(j+1\right)\right)+1,\;j\in \{1,2,\ldots ,12\times len-1\}$$

(5) $$mat^{n}\left(k,\;1:12\right)=\frac{mat\left(k,\;1:12\right)}{sum\left(mat\left(k,\;1:12\right)\right)+\varepsilon },\;k\in \{1,2,\ldots ,12\}$$

(6) $$fv\left(u\right)=mat^{n}\left(k,l\right),\;l\in \left\{1,2,\ldots ,12\right\},\;u\in \left\{1,2,\ldots ,144\right\}$$

Herein, mat: transition matrix, sum: summation function and fv: feature vector with a length of 144. Herein, we have applied row-based normalization to the transition matrix and we obtained $mat^{n}$ to create feature vector.

Step 3: Repeat Steps 1–2 to create the feature matrix (X) until all ECG leads are included.

#### 2.2.2. Feature Selection

This phase is one of the most critical phases of the presented model since both classification and explainable results are obtained by utilizing the selected features. To select the most informative features, the Iterative Neighborhood Component Analysis (INCA) feature selector has been used, which was proposed by Tuncer et al. in 2020 [37]. INCA is specifically designed to enhance the selection of features that contribute most significantly to the model’s performance, thus improving the overall accuracy and interpretability of the results. The effective use of INCA ensures that the model focuses on the most relevant data, reducing complexity while maintaining or even increasing the classification performance. In this feature selector, the qualified indexes are generated using the NCA feature selector. A range of features is then selected by the user, and iterative feature selection is performed. During this iterative process, the misclassification rate for each selected feature vector is computed. In the final step, the feature vector with the minimum misclassification rate is chosen. The steps of the INCA feature selector are outlined below to ensure that only the most informative and relevant features are retained for further analysis and classification. This approach allows for a more refined model that is both accurate and interpretable, focusing on the features that contribute most significantly to the prediction task.

Step 4: Normalize each feature vector.

(7) $$X^{n}\left(:,i\right)=\frac{X\left(:,i\right)-\min\left(X\left(:,i\right)\right)}{max(X\left(:,i\right))-\min\left(X\left(:,i\right)\right)+\varepsilon }$$

Herein, $X^{n}$: the normalized feature matrix using min-max normalization. The NCA feature selector is a distance-based feature selector. Therefore, we have applied

Step 5: Generate the qualified indexes of each feature deploying NCA.

(8) $$idx=NCA(X^{n},y)$$

Herein, NCA: NCA feature selector and y: actual outcome.

Step 6: Select the features iteratively and compute the misclassification rate of each selected features deploying the kNN classifier.

(9) $$sf^{j}\left(d,g\right)=X^{n}\left(d,idx\left(g\right)\right),\;j\in \left\{1,2,\ldots ,144\right\},\;g\in \left\{1,2,\ldots ,j\right\},\;n\in \left\{1,2,\ldots ,NE\right\}$$

(10) $$loss\left(j\right)=kNN\left(sf^{j},y\right)$$

Herein, sf: selected feature vector, NE: number of ECG signals, loss: misclassification rate and kNN: kNN classifier [38].

Step 7: Choose the optimal feature vector with greedy algorithm.

(11) ind=argmin(loss)

(12) $$sel=sf^{ind}$$

Herein, sel: the final selected feature vector.

#### 2.2.3. Classification

In the classification phase, the proposed model employs the commonly known distance-based classifier, specifically the kNN classifier. This classifier is widely used in pattern recognition and machine learning due to its simplicity and effectiveness. The steps for the classification process in the proposed model are as follows:

Step 8: Classify the selected feature by deploying the kNN classifier.

(13) pred=kNN(sel,y)

Herein, pred: the predicted output.

The proposed Cardioish-based XFE model effectively extracts distinctive features using a basic kNN classifier. The lead-based transformation and transition table-based feature extractor demonstrate their effectiveness when combined with a simple classifier. Additionally, the best results are achieved with the INCA feature selector, which, like kNN, is a distance-based feature selection method.

#### 2.2.4. Explainable Results Generation with Cardioish

**Figure 2:**
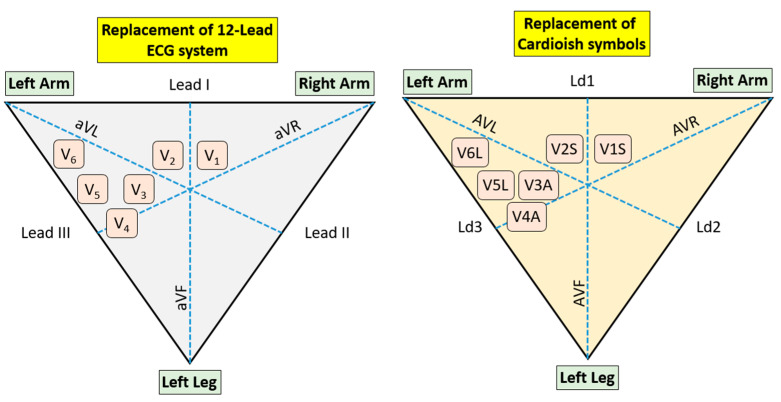
The replacement of the 12-leads and the proposed Cardioish symbols.

To generate explainable results, we have introduced a new symbolic language called Cardioish. This language is designed to provide a clear and interpretable representation of ECG data by assigning specific symbols to each lead involved in the signal processing. The graphical representation of how each lead corresponds to a symbol in Cardioish is shown in Figure 2. This visual tool aids in understanding the relationship between the leads and their respective symbols, facilitating the interpretation of ECG signals through an easily understandable symbolic system.

Cardioish is a symbolic language developed to make ECG signal interpretation more accessible and explainable, especially in integrating connectome theory into cardiology. Each of the 12 symbols in Cardioish corresponds to a specific ECG lead, each representing different parts of the heart’s electrical activity.

Symbols and Their Meanings:

Ld1 (Lead I): Focuses on the left atrium and left ventricle, useful for detecting atrial arrhythmias and left atrial enlargement.

Ld2 (Lead II): Monitors general heart rhythm, particularly the P wave, and is crucial for identifying atrial depolarization and sinus arrhythmias.

Ld3 (Lead III): Assesses the heart’s inferior wall, often used for diagnosing inferior myocardial infarctions (MIs).

AVR: Reflects the right ventricle’s activity and the heart’s base, useful in detecting severe global ischemia or improper lead placement.

AVL: Monitors the left ventricular lateral wall, important for diagnosing lateral MIs.

AVF: Focuses on the inferior wall of the left ventricle, aiding in the detection of inferior MIs.

V1S (Lead V1): Concentrates on the right ventricle and interventricular septum, essential for assessing right ventricular hypertrophy and septal infarctions.

V2S (Lead V2): Assesses the anterior and septal regions, important for diagnosing anterior and septal MIs.

V3A (Lead V3): Monitors the anterior wall and septum, sensitive to changes in the anterior wall, helping to identify anterior MIs.

V4A (Lead V4): Focuses on the lower part of the left ventricle, crucial for diagnosing anterior MIs.

V5L (Lead V5): Monitors the lateral wall of the left ventricle, playing a critical role in lateral MI diagnosis.

V6L (Lead V6): Focuses on the lower-lateral wall of the left ventricle, crucial for lateral MI diagnosis.

These symbols form the basis of Cardioish, a language designed to interpret ECG signals using a connectome graph, which visually represents the heart’s electrical activity and its association with various cardiac conditions. This approach aims to provide more explainable and interpretable results in cardiology by aligning ECG signal analysis with connectome theory.

Step 9: Create Cardioish symbol by using the indexes of the selected features.

(14) C={Ld1, Ld2,Ld3,AVR,AVL,AVF,V1S,V2S,V3A,V4A,V5L,V6L}

(15) $$x_{1}=\left\lceil \frac{sind(h)}{12}\right\rceil ,\;h\in \left\{1,2,\ldots ,nosf\right\}$$

(16) $$x_{2}=sind\left(h\right)\left(mod\;12\right)+1$$

(17) $$sent\left(w\right)=C\left(x_{1}\right),\;w\in \left\{1,3,\ldots ,2\times nosf-1\right\}$$

(18) $$sent\left(w+1\right)=C\left(x_{2}\right)$$

(19) $$sind\left(w\right)=x_{1}$$

(20) $$sind\left(w+1\right)=x_{2}$$

Herein, C: Cardioish symbol array, sind: indices of the selected features, nosf: number of the selected features, sent: the created Cardioish sentence and sind: indexes of the symbols.

Step 10: Compute the probability of each symbol to calculate the entropy value of the generated sentence.

Step 11: Create the connectome graph by deploying the transition table of the symbols in the generated Cardioish sentence.

The 11 steps given above have been defined in the presented Cardioish-based XFE model.

## 3. Performance Evaluation

In this section, the computed explainable and classification results are presented. The proposed model was implemented using MATLAB (version 2024a) on a laptop equipped with a 3.2 GHz central processing unit (CPU), 32 GB of main memory, and running Windows 11 as the operating system.

The parameters used for the proposed Cardioish-based XFE model are outlined in Table 1.

In this research, two datasets were used to obtain results: (i) the MI classification dataset and (ii) the mental disorder detection dataset. To evaluate the performance of these datasets, classification accuracy and geometric mean performance metrics were employed. Additionally, 10-fold cross-validation was utilized for validation.

**Figure 3:**
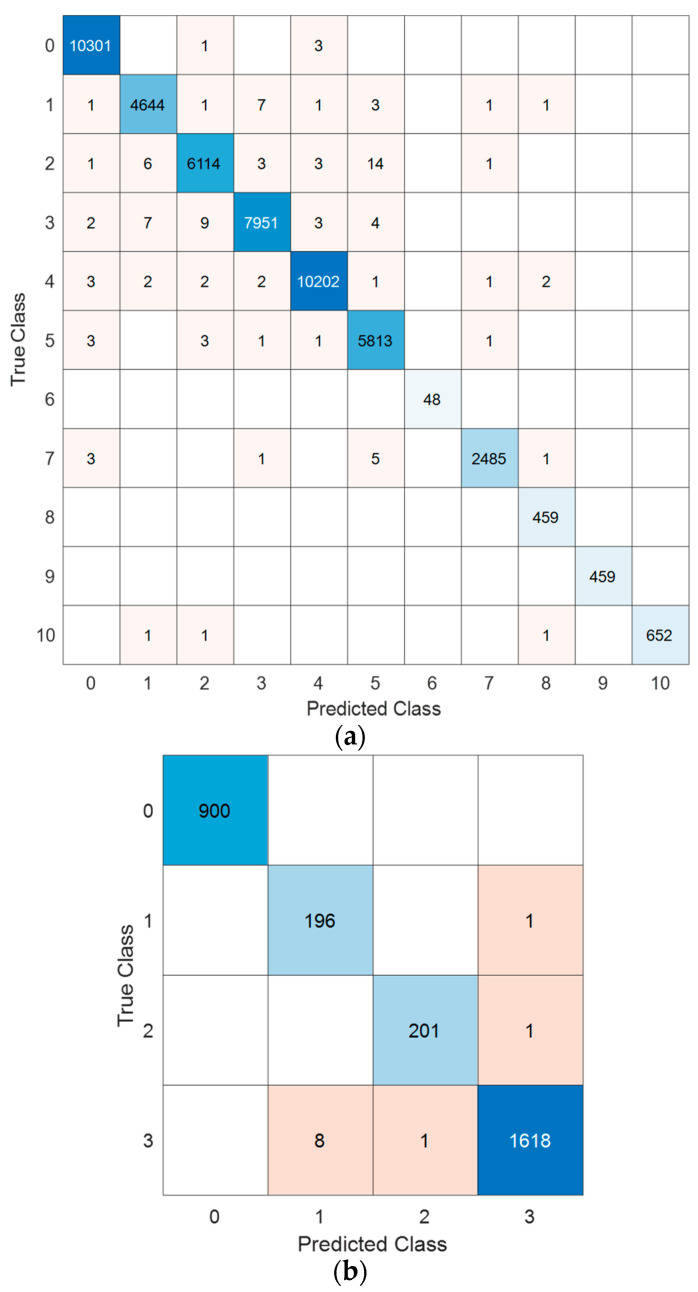
The computed confusion matrices of the proposed Cardioish-based XFE model on the MI and mental disorder detection datasets. (**a**) MI detection. Here, 0: Normal, 1: Anterior, 2: Anterior Lateral, 3: Anterior Septal, 4: Inferior, 5: Inferior Lateral, 6: Inferior Posterior, 7: Inferior Posterior Lateral, 8: Lateral, 9: Posterior and 10: Posterior Lateral. (**b**) Mental disorder detection dataset. Here, 0: Normal, 1: Bipolar, 2: Depression, 3: Schizophrenia.

The computed results are presented in Table 2, and the confusion matrices of the proposed model for both datasets are shown in Figure 3.

The computed results are demonstrated in Table 2, based on the confusion matrices obtained.

As shown in Table 2, the proposed model achieved over 99.5% classification accuracy and geometric mean for both datasets. The explainable results have also been presented using the proposed model. First, the model generates a Cardioish sentence, followed by numerical analysis of this sentence. Additionally, connectome diagrams for these datasets have been generated. The generated XAI results are demonstrated below.

Based on the analysis of Table 3, the following interpretations for the MI and mental disorder datasets are provided:

MI Dataset:

The patterns identified in the Cardioish sentence generated from the MI dataset can be instrumental in refining algorithms within AI-driven ECG analysis tools. Notably, the emphasis on the V3S and V4S leads could be leveraged to develop specific markers or triggers for the early detection of anterior myocardial infarctions (MIs), a condition that often necessitates prompt medical intervention. The identification of these specific leads highlights their critical role in monitoring the anterior region of the heart, where early detection of ischemic changes can significantly impact patient outcomes.

Mental Disorder Detection Dataset:

Depression: The frequent utilization of lateral and anterior leads, such as V5S, V3S, and V4S, may suggest a reduction in heart rate variability, a characteristic commonly observed in depressive states. This consistent lead involvement might reflect a stable yet abnormal autonomic tone, indicative of an underactive parasympathetic nervous system often associated with depression.

Bipolar Disorder: The erratic transitions observed between leads could be indicative of the alternating manic and depressive states characteristic of bipolar disorder. The frequent engagement of AVR and V1S leads might point to the detection of fluctuations in autonomic control, which are hallmarks of this condition.

Schizophrenia: In schizophrenia, the Cardioish sentence reveals more irregular and varied transition patterns, particularly in the high frequency and transitions of the AVR and Ld3 leads. These patterns could represent a dysregulated autonomic response, a common feature in schizophrenia that can increase cardiovascular risk. The complexity and irregularity in the lead transitions may correspond to the diverse and often disorganized symptoms of schizophrenia, affecting multiple systems simultaneously.

Normal: The absence of abrupt or erratic patterns, coupled with a balanced distribution across leads, typically suggests normal autonomic regulation. This pattern indicates no significant abnormalities in heart function, which is expected in the control group.

**Figure 4:**
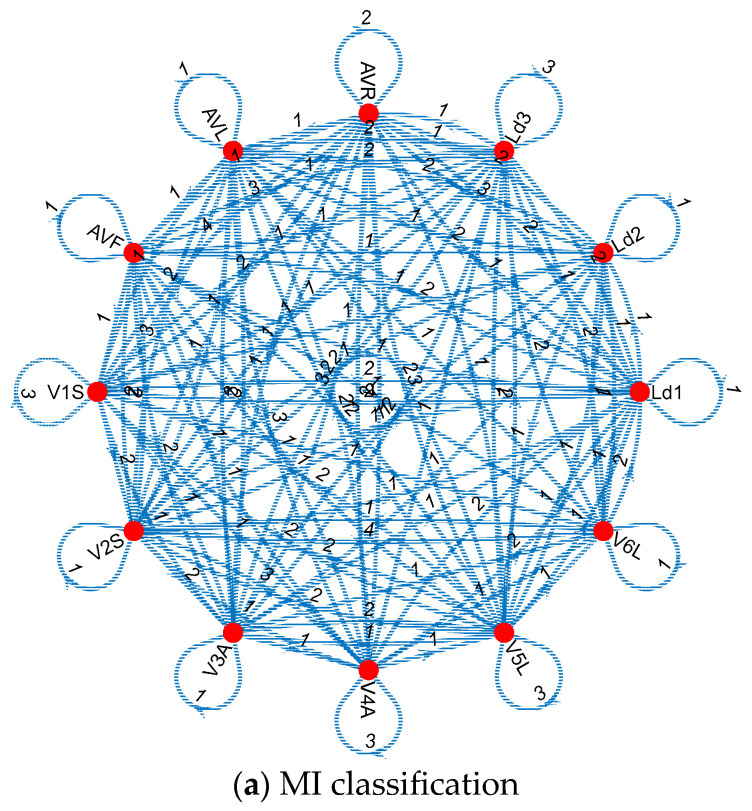

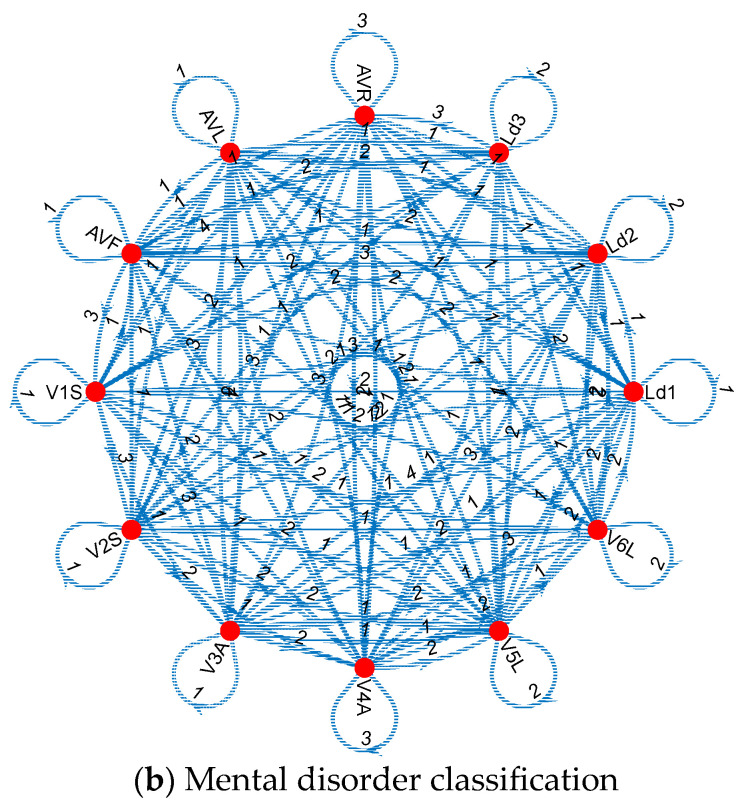
The generated connectome graphs. The number indicates the count of transitions.

Moreover, the generated connectome graphs, which visually represent these Cardioish sentences, provide a deeper understanding of the lead interactions and the spatial distribution of electrical activity across the heart. These graphs further emphasize the regularity in MI-related cases versus the irregularity in mental disorders, offering critical insights for differential diagnosis and targeted interventions. The generated connectome graphs generated for these datasets are also shown in Figure 4.

The findings obtained using the above connectome graphs (see Figure 4) are as follows. The irregularity of the transition tables related to mental disorders indicates that autonomic nervous system dysfunction is widespread in these diseases and has a direct or indirect effect on heart rhythm. This finding emphasizes the need to manage the cardiovascular risks of patients with mental disorders. The regularity and localization of the transition tables related to MI indicate that the heart damage in this disease is related to a specific region and that this region is evident in certain ECG leads. This plays a critical role in the early diagnosis and treatment of MI.

Moreover, we have computed the entropy values of these Cardioish sentences obtained from MI and mental disorder classification datasets, and the computed results are demonstrated in Table 4.

The entropies clearly demonstrate that both Cardioish models are complex, given the presence of 11 and 4 classes in these datasets. However, the mental disorder classification is more complex than MI detection when using ECG signals.

## 4. Discussion

In this research, we presented a new symbolic language called Cardioish, along with a Cardioish-based XFE model. Our proposed XFE model delivered both classification and explainable results. To demonstrate the high classification capability of the proposed model, we utilized two publicly available datasets. The Cardioish-based XFE model achieved classification accuracies of 99.78% on the MI dataset and 99.62% on the mental disorder classification dataset. These results clearly highlight the strong classification ability of the proposed model. The introduced Cardioish-based XFE model achieves high classification performance by combining various metrics to address potential overfitting concerns. The presented Cardioish-based XFE model uses 10-fold cross-validation (CV) to validate its performance across different subsets of the data, minimizing the risk of overfitting to the training dataset. Additionally, the INCA feature selector aims to extract only the most relevant features and achieve the highest accuracy with the fewest features, reducing noise and redundancy. By utilizing a simple kNN classifier, which is less prone to overfitting compared to complex models, the Cardioish-based XFE model achieves even more robust generalization. Performance metrics derived from two independent datasets also demonstrate the model’s high generalizability. Moreover, Cardioish and connectome diagrams provide transparency.

Additionally, a comparative results table is provided in this section to position our model within the existing literature. The detailed results are presented in Table 5.

Our proposed XFE model delivered both classification and explainable results. To demonstrate the high classification capability of the model, we utilized two publicly available datasets. The Cardioish-based XFE model achieved classification accuracies of 99.78% on the MI dataset and 99.62% on the mental disorder classification dataset, respectively. These results clearly highlight the robust classification ability of the proposed model. Additionally, a comparative results table is provided in this section to position our model within the existing literature. Furthermore, the presented model has linear time complexity and is competitive with deep learning models, whereas deep learning or optimization-based models have an exponential time burden. In this aspect, the presented model is a highly accurate, interpretable, and lightweight ECG classification model. The results are detailed in Table 5.

**Figure 5:**
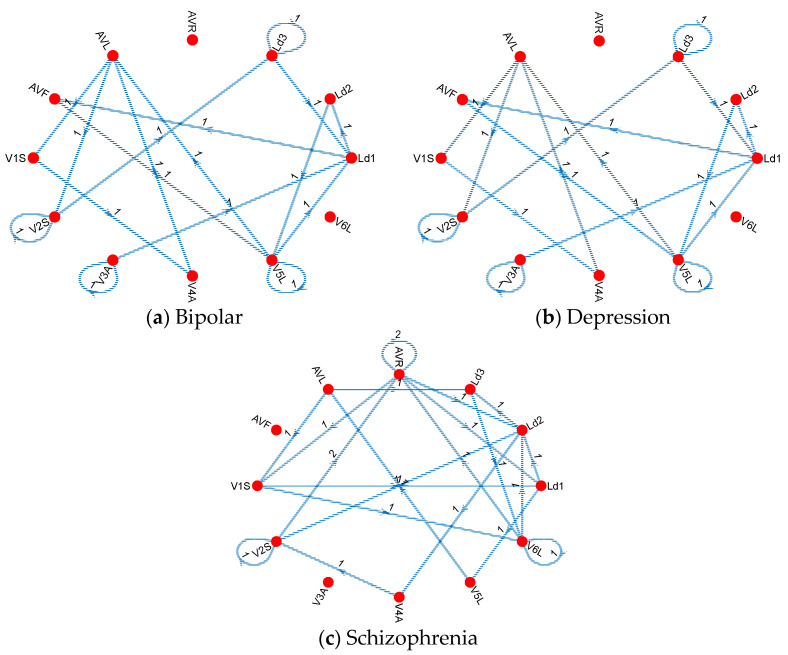
The connectome graphs of the mental disorders were utilized in this study. Herein, the computed transition table related to mental disorders is defined. Figure 5 illustrates the cardiac connectome diagrams for (**a**) Bipolar disorder, (**b**) Depression, and (**c**) Schizophrenia detection. The red circles represent Cardioish symbols, while the edges depict the connections between these symbols. The numbers indicate the counts of the transitions.

The smaller dataset used in this study is the mental disorder detection dataset, which includes three disorders: Bipolar, Depression, and Schizophrenia. To extract the connectome graphs for these disorders, we analyzed Bipolar vs. Control, Depression vs. Control, and Schizophrenia vs. Control cases. The resulting connectome graphs are illustrated in Figure 5.

**Figure 6:**
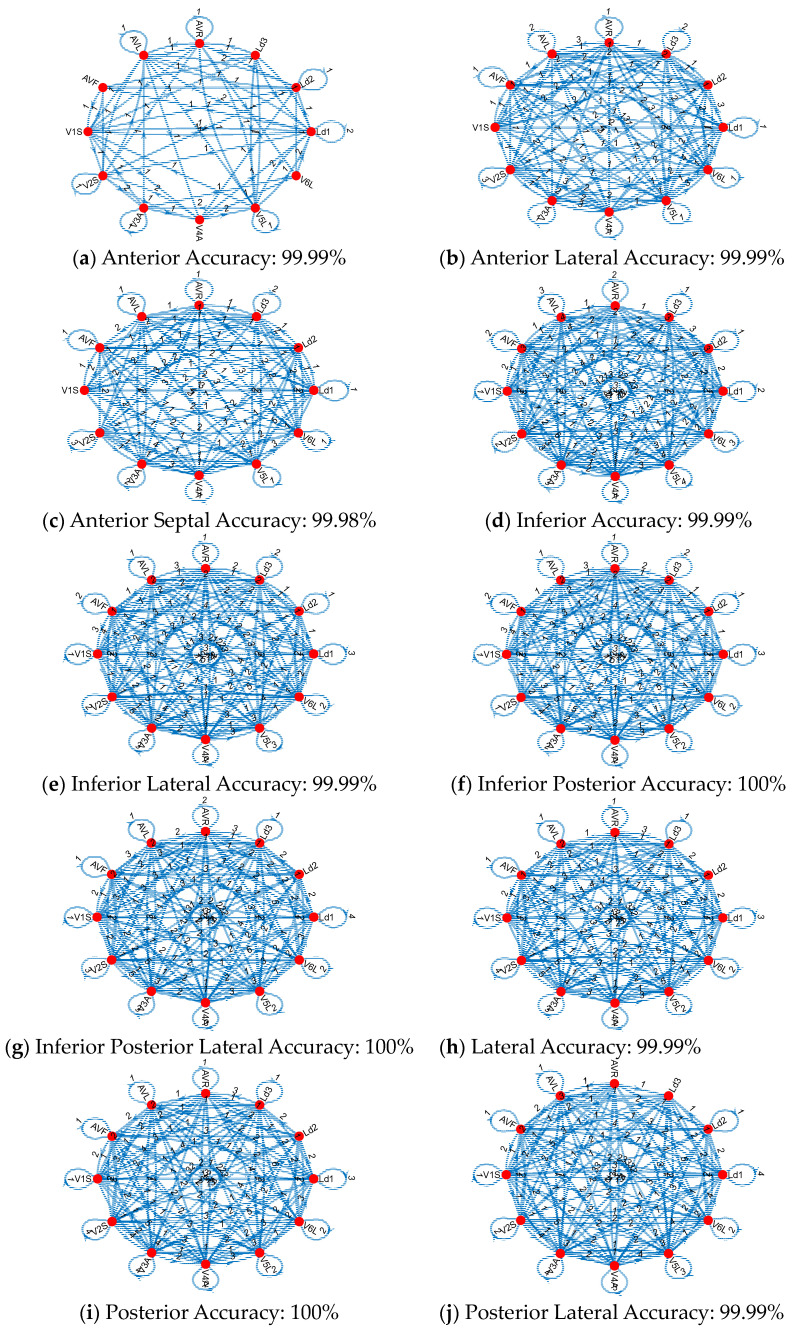
The identical connectome graphs of the MIs. Herein, the computed transition table related to MIs are depicted using graphical demonstration to obtain cardiac connectome diagram. Figure 6 showcases the cardiac connectome diagrams for (**a**) Anterior MI, (**b**) Anterior Lateral MI, (**c**) Anterior Septal MI, (**d**) Inferior MI, (**e**) Inferior Lateral MI, (**f**) Interior Posterior MI, (**g**) Interior Posterior Lateral MI, (**h**) Lateral MI, (**i**) Posterior MI and (**j**) Posterior Lateral MI detection. The red circles represent Cardioish symbols, while the edges depict the connections between these symbols. The numbers indicate the counts of the transitions.

The connectome graph (Figure 5) for bipolar disorder, depression, and schizophrenia shows distinct patterns of connections and transitions between various symbols (corresponding to different ECG leads or regions). According to the obtained connectome diagrams, the findings are as follows: The connections in the bipolar disorder graph (Figure 5a) are relatively spread out and have a more evenly distributed transition pattern between the nodes. This suggests that bipolar disorder may have a more general or widespread effect on the electrical activity of the heart. The presence of more than one bidirectional arrow between the nodes suggests cyclical or fluctuating activity that may reflect the alternating phases of mania and depression in bipolar disorder. The depression graph (Figure 5b) showcases denser connections between certain nodes, especially Ld3, V3A, and V5L. This suggests that depression has a more localized effect on certain regions of the heart’s electrical activity. The connectome graph (Figure 5c) for schizophrenia shows a complex connection pattern with several nodes showing multiple transitions, such as AVR and Ld2. The presence of more complex interactions and several nodes with higher connectivity may indicate the widespread and multifaceted nature of the effects of schizophrenia on the autonomic nervous system and cardiac function, or may indicate a cardiac effect of schizophrenia medications. This complexity may correspond to the diverse and often disorganized symptoms associated with schizophrenia, which may affect multiple systems simultaneously. In the MI dataset, there are 11 classes, including 1 control and 10 MI types. In this phase, to extract the specific graphs of the MIs, we defined 10 cases, which include the MI type and control classes, to show MI connectome graphs. These graphs are demonstrated in Figure 6.

Figure 6 showcases that all MIs have unique connectome graphs. By using the proposed Cardioish language, the connectome graphs for these MIs have been generated, making the MI detection process easier. The provided connectomes in Figure 6 are used to visualize the generated Cardioish sentences. In this aspect, these diagrams have showcased the transition of the ECG leads.

Cardio connectome/Cardiac connectome diagrams have been proposed in cardiology to visualize and understand the complex electrical interactions in the heart. Connectome diagrams are commonly associated with neuroscience, where they map the neural connections in the brain. In this article, cardio connectome diagrams are adapted to show the relationships between the 12 leads of an ECG and the electrical activity of the heart, as cardiac diseases have the highest mortality rate. The presented cardiac connectome diagrams provide us with insights into the structure and condition of the heart.

Creating cardiac connectome diagrams involves converting raw ECG signals into symbolic representations using the Cardioish language. Cardioish assigns specific symbols to each ECG lead, effectively converting the complex electrical signals into a sequence of symbols, or a “Cardioish sentence.” Transition tables are then used to map the transitions between these symbols and capture the dynamic interaction of electrical impulses as they propagate through the chambers of the heart. This process converts high-dimensional, time-series ECG data into a structured format suitable for graphical display and analysis. For example, leads II, III, and aVF focus on the lower part of the heart, while leads V1 and V2 provide information about the septum region. By integrating data from all leads, connectome diagrams provide the best model of how electrical signals flow through various parts of the heart during each cardiac cycle, providing crucial insights into detecting abnormalities.

Connectome diagrams allow patterns to be identified that are associated with specific cardiac conditions, and we have demonstrated these in this article. By comparing connectome diagrams of healthy individuals with those of patients who exhibit cardiac abnormalities, researchers can identify trends and characteristics that indicate specific conditions, thus providing more information from the ECG data.

Cardioish and connectome diagrams can also help simplify complex and difficult-to-interpret ECG data, helping to quickly identify important patterns without being overwhelmed by the details of ECG waveforms. They also facilitate communication between medical professionals by providing a clear depiction of cardiac electrical activity.

Connectome diagrams can distinguish between local abnormalities and general dysfunctions by mapping the transitions between channels. For example, a blockage in a particular coronary artery may only affect certain regions of the heart, resulting in local changes in the connectome diagrams.

Incorporating connectome diagrams into machine learning models will improve both performance and interpretability and will also make machine learning methods more cardiologically meaningful.

By producing diagrams at different stages of a disease or before and after treatment interventions, it is also possible to visualize improvements or deterioration in the electrical activity of the heart. This capability provides valuable insights for evaluating the effectiveness of treatments, adjusting therapies as needed, and providing patients with tangible evidence of their progress.

Each cardiac condition can produce a unique connectome diagram that effectively acts as a fingerprint for that condition. Alternatively, a dictionary of these connectomes can be created that can be easily read by non-cardiologists.

The recommended Cardioish-based XFE models clinical applications have been given below with two cases.

Case Study 1: A 58-year-old male patient presents to the clinic with chest discomfort, and ECG data is obtained. The ECG data is processed using the Cardioish-based XFE model, which produces a Cardioish sentence highlighting significant transitions involving leads V1S, V2S, and V3A. The connectome plot shows connections concentrated in the anterior leads.

Interpretation: The physician interprets these findings as indicative of an anterior myocardial infarction. The visual representation aids in rapid decision-making and prompts immediate intervention.

Case Study 2: A 43-year-old female patient with a history of depression presents to the psychiatric ward reporting palpitations. The model analyzes the ECG data to produce a Cardioish sentence with irregular patterns and a connectivity plot showing widespread irregular transitions.

Interpretation: A psychiatrist identifies patterns associated with autonomic dysfunction linked to depressive disorders. This finding enables the interpretation of mental health conditions from a cardiac perspective.

Integration into Clinical Practice: Physicians can gain an intelligent assistant by incorporating the Cardioish model into their diagnostic toolkit through learning the symbolic language, which simplifies complex ECG data into understandable patterns. Additionally, the creation of the Cardioish dictionary and its integration into the digital environment will increase the speed of clinicians’ case resolution and improve accurate diagnosis rates.

Advantages, limitations and future work have also been discussed below.

Advantages:

- The presented Cardioish-based XFE model achieved over 99% accuracy in both MI and mental disorder classification, highlighting its effectiveness in clinical applications.
- By introducing the Cardioish language, the model not only classifies but also provides explainable results, which are crucial for clinical decision-making.
- Our model effectively bridges cardiology with connectome theory, offering a new perspective on understanding the cardiac effects of various diseases. The advantages of the connectome diagrams are as follows:
  ⚬ By using connectome diagrams, cardiac functions have been effectively mapped.
  ⚬ ECG signals have 12 leads, and these signals involve complex interactions. Using Cardioish and Cardioish-based connectome diagrams, the meaning of these complex interactions has been extracted.
  ⚬ Cardiac connectome diagrams make it easier to understand specific patterns and abnormalities.
  ⚬ These cardiac diagrams can be utilized as XAI tools and features in machine learning models to achieve high classification performance.
  ⚬ By integrating connectome theory, commonly used in neuroscience, with cardiology, researchers may gain new insights into how systemic disorders, such as diabetes, stress, or mental health problems, affect heart function.
  ⚬ These connectomes can also be used to monitor the progression of cardiac disorders and the efficiency of treatments.
- The recommended lead transformation and transition table-based feature extraction methods successfully captured essential patterns in ECG signals.
- The presented model generates unique connectome diagrams for each case. In this aspect, the recommended Cardioish model simplifies the disorder detection processes.

Limitations:

- While the Cardioish language provides explainable results, the interpretation of these results might still be complex for practitioners without a deep understanding of the symbolic language.

Future works:

- The proposed Cardioish-based XFE model will be applied to more ECG signal datasets to develop a comprehensive Cardioish dictionary.
- Tools and guidelines for using Cardioish will be prepared for students and professionals to enhance its accessibility.
- The feasibility of real-time application of Cardioish-based models in clinical settings will be investigated.

## 5. Conclusions

In this study, the Cardioish-based XFE model was introduced for the classification and interpretation of ECG signals. The model demonstrated high classification accuracy, achieving 99.78% on the MI dataset and 99.62% on the mental disorder detection dataset. These results highlight the model’s effectiveness in accurately identifying various cardiac conditions.

Moreover, the Cardioish language provided a novel approach to generating explainable results, offering a symbolic representation of ECG data that aligns with connectome theory. This integration was further enhanced by the generation of graph-based hash codes using Cardioish symbols, allowing for the creation of connectome graphs that visually represent the interactions and transitions between different ECG leads. These graphs not only offer insights into the cardiac conditions being analyzed but also enable the generation of unique identifiers for specific cardiac states through the hash codes derived from the connectome graphs.

While the obtained high classification performances for both ECG datasets are impressive, it is important to contextualize them within the broader landscape of ECG analysis research, where numerous studies have reported comparable or even superior performance using various machine learning and deep learning methodologies. However, the Cardioish model differentiates itself from other ECG classification models through its emphasis on interpretability. By incorporating the Cardioish symbolic language and connectome diagrams, the model not only delivers robust classification performance (using two separate multi-lead datasets in this paper) but also provides interpretable results that are essential for clinical decision-making. Additionally, the computational efficiency of the model, achieved through the use of a basic kNN classifier, demonstrates its suitability for real-time applications and resource-constrained environments. Furthermore, the integration of connectome theory introduces a new framework for visualizing and understanding the complex electrical interactions within the heart, enhancing both diagnostic accuracy and clinical insights. The classification performance was also used to validate the accuracy of the connectome diagrams and the resulting Cardioish strings. These features collectively make the Cardioish model a valuable tool in cardiology.

## Figures and Tables

**Table 1:** The parameters of the methods used.

Phase	Method	Parameters
Feature extraction	Lead-transformer	Sorting criteria: Descending, Length of the transformed signal: 12x
	Transition table-based feature extractor	Length of the features: 144, Normalization: Row-based.
Feature selection	INCA	Start value: 1, Stop value: 144, Loss generation function: kNN, Optimal feature selection function: Greedy function based on minimum loss value
Classification	kNN	k: 1, Distance: L1-norm, Validation: 10-fold CV
XAI generation	Cardioish	Number of symbols: 12, XAI generation: Entropy and transition table-based connectome graph generation.

**Table 2:** The results (%) of the proposed model for the datasets used.

Performance Metric	MI	Mental Disorder Detection
Classification accuracy	99.78	99.62
Geometric mean	99.79	99.61

**Table 3:** The Cardioish sentences generated for MI and mental disorder dataset.

XAI	MI Dataset	Mental Disorder Dataset
Cardioish sentence	AVLV1SV2SAVRAVRLd2Ld2AVLV3AV4AV6LV4AV5LLd1V3AV1SV2SV2SV5LV3AV4AV2SV4ALd1V6LV3ALd1V5LLd3AVFAVFLd1AVFV2SLd1Ld2V6LV6LAVLV4AAVRAVFLd2V4AV4AV4AV4AAVRV1SLd2Ld1V3AAVLV2SV3AV6LV2SV3AV6LAVRV1SLd3AVRAVRV5LV1SV5LV2SAVLLd3AVRV4ALd1V2SAVLAVLLd2Ld2AVFV1SLd2V2SLd3AVRV2SAVLLd3V2SLd2V5LV5LAVRV3AV5LLd1AVFLd2AVRAVRV1SAVFLd3V6LV1SLd3V6LV1SV4ALd1AVRV3AAVRLd1Ld1V6LAVLV3AAVLV3ALd2V3ALd1V3AV3AAVRV3AAVRV2SV5LLd2V4AV6LLd2V6LLd1AVLV4AAVLAVFLd2V4AV5LAVLLd2V5LAVFV1SV1SLd1Ld3V5LV5LV6LLd1V4ALd3AVFAVRLd3V4AV2SLd3Ld3Ld3AVLV3AV1SLd1V3AAVFAVLV6LV6LLd2V1SV2SLd2V1SLd2V3AAVFV5LAVLV5LV1SV5LV4AAVFV1SAVRV1SV3AV1SV6L	V4AV2SV5LV3AAVLV1SV2SV2SV6LV4AV4AV4AAVFAVRLd2V6LV3AV1SLd1V5LV5LV5LLd2AVLV2SV3AAVFLd3V1SLd3AVLV2SV6LV6LLd1AVLV3AV4AV2SV4ALd1Ld2Ld2V5LV3AV3ALd3V4AV4AAVLV4AAVRV1SLd2AVLAVLV2SAVLAVFLd1Ld3AVFAVRLd2AVRAVFV2SV5LAVLV4AAVRAVRV5LV1SV1SV1SAVLLd3Ld3V5LV2SAVRV5LAVLV6LLd1AVRAVLV4AV1SAVRV3AV5LLd3V2SLd3Ld3AVRAVRV2SLd3V6LAVFV2SLd1Ld1V4AV5LAVFAVFV3AAVLV5LV2SV3AAVRLd3Ld1AVFV4ALd1Ld3V6LLd3V3ALd1AVLLd2Ld1V4AAVLLd1V1SV3AAVLV3ALd2AVRV1SV2SV6LLd2AVFV5LV5LLd1V6LV2SAVFV3AAVRV5LV1SAVLLd3V3AAVRV4AV2SV6LV1SLd1V2SAVFAVRV1SV1SV5LAVFV1SV5LV6LLd3Ld3V5LAVRLd2V2SLd2V3ALd3AVLLd1AVRV1SAVRLd1V1S
Frequency	Ld1: 16, Ld2: 18, Ld3: 13, AVR: 18, AVL: 16, AVF: 13, V1S: 18, V2S: 15, V3A: 19, V4A: 17, V5L:16, V6L:15	Ld1: 16, Ld2: 11, Ld3: 17, AVR: 19, AVL: 18, AVF: 13, V1S: 17, V2S: 18, V3A: 15, V4A: 15, V5L:18, V6L:11

**Table 4:** Information entropies of the generated Cardioish sentences.

Dataset	Information Entropy	Maximum Entropy
MI	3.5637	3.5850 ($=\log_{2}\left(12\right)$)
Mental disorder classification	3.5752	3.5850 ($=\log_{2}\left(12\right)$)

**Table 5:** Comparative results (%) for same datasets.

Study	Method	XAI	Performance Measurements	Time Burden
MI dataset				
Martin et al. [39]	Long short-term memory neural network	No	Acc: 91.36	Exponential
Pal et al. [40]	CardioNet	No	Acc: 98.92	Exponential
Hernandez et al. [41]	Recurrent neural networks and distribution parameters	No	Acc: 97.40 Sen: 94.70 Spe: 100.0	Exponential
Kolhar et al. [42]	i-AlexNet Architecture	No	Acc: 98.20 Pre: 97.50 Rec: 97.20 F1: 97.90	Exponential
Our study	Cardioish-based XFE model	Yes	Acc: 99.78 Gm: 99.79	Linear
Mental disorder dataset				
Tasci et al. [36]	Ternary pattern	No	Acc: 96.25	Linear
Khare et al. [20]	Optimized hybrid ensemble model	No	Acc: 98.15	Exponential
Our study	Cardioish-based XFE model	Yes	Acc: 99.62 Gm: 99.61	Linear

## Data Availability

The authors are committed to making the data available if requested by the Journal.
